# Mapping the primate thalamus: systematic approach to analyze the distribution of subcortical neuromodulatory afferents

**DOI:** 10.1007/s00429-023-02619-w

**Published:** 2023-03-08

**Authors:** Isabel Pérez-Santos, Miguel Ángel García-Cabezas, Carmen Cavada

**Affiliations:** 1 Department of Anatomy, Histology and Neuroscience, School of Medicine, Facultad de Medicina, Universidad Autónoma de Madrid, Calle Arzobispo Morcillo 4, 28029 Madrid, Spain; 2 PhD Program in Neuroscience, Universidad Autónoma de Madrid-Cajal, Madrid, Spain; 3 Neural Systems Laboratory, Department of Health Sciences, Boston University, Boston, MA USA

**Keywords:** Thalamus, Thalamic nuclei, Acetylcholine, Dopamine, Adrenaline, Noradrenaline, Serotonin, Histamine

## Abstract

Neuromodulatory afferents to thalamic nuclei are key for information transmission and thus play critical roles in sensory, motor, and limbic processes. Over the course of the last decades, diverse attempts have been made to map and describe subcortical neuromodulatory afferents to the primate thalamus, including axons using acetylcholine, serotonin, dopamine, noradrenaline, adrenaline, and histamine. Our group has been actively involved in this endeavor. The published descriptions on neuromodulatory afferents to the primate thalamus have been made in different laboratories and are not fully comparable due to methodological divergences (for example, fixation procedures, planes of cutting, techniques used to detect the afferents, different criteria for identification of thalamic nuclei…). Such variation affects the results obtained. Therefore, systematic methodological and analytical approaches are much needed. The present article proposes reproducible methodological and terminological frameworks for primate thalamic mapping. We suggest the use of standard stereotaxic planes to produce and present maps of the primate thalamus, as well as the use of the Anglo-American school terminology (vs. the German school terminology) for identification of thalamic nuclei. Finally, a public repository of the data collected under agreed-on frameworks would be a useful tool for looking up and comparing data on the structure and connections of primate thalamic nuclei. Important and agreed-on efforts are required to create, manage, and fund a unified and homogeneous resource of data on the primate thalamus. Likewise, a firm commitment of the institutions to preserve experimental brain material is much needed because neuroscience work with non-human primates is becoming increasingly rare, making earlier material still more valuable.

## Introduction

The thalamus is a nuclear complex in the diencephalon, whose neurons participate in sensory, motor, and limbic functions. These functions are supported by bidirectional connections with the cerebral cortex and a variety of subcortical afferents from sensory pathways, globus pallidus, cerebellum, amygdala, and the hypothalamus. The information carried by the subcortical afferents, as well as from cortical layers V and VI, is processed, modulated, integrated, and transmitted to the cerebral cortex by thalamocortical projection neurons (Jones 2007).

The thalamus receives cortical afferents, which are glutamatergic, and subcortical afferents, which are either glutamatergic (for example, from the cerebellum or the somatosensory gracile and cuneate nuclei), GABAergic (for example, from the internal globus pallidus or the *substantia nigra reticulata*), as well as cholinergic, adrenergic, noradrenergic, dopaminergic, serotoninergic, histaminergic, and peptidergic. This review focuses on the cholinergic and aminergic afferents, which are functionally modulatory; they shall be referred to as “neuromodulatory afferents” throughout the text, to distinguish them from glutamatergic (excitatory) and GABAergic (inhibitory) afferents, as well as from other modulatory agents such as circulating hormones or extracellular space compounds. We consider that the term neuromodulatory conveys a more specific meaning that other terms, such as “non-specific afferents”, which was used by Jones (2007). Subcortical neuromodulatory afferents reach the thalamus from the brainstem, the basal prosencephalon, and the hypothalamus and play important roles in the modulation of sensory, motor, limbic, and cognitive circuits at the thalamic level (Jones 2007; Varela 2014).

The study of neuromodulatory afferents in brain structures (the thalamus in the present case) involves mapping and quantifying the distribution of several molecules across brain tissue, including neurotransmitter synthetizing enzymes—for example, choline acetyltransferase (Steriade et al. 1988; Heckers et al. 1992; Rico and Cavada 1998b); neurotransmitter transporters—for example, vesicular transporter of acetylcholine (Rico and Cavada 1998b); neurotransmitter degradation enzymes—for example, acetylcholinesterase (Cavada et al. 1995); and neurotransmitter receptors—for example, cholinergic nicotinic receptors (Cimino et al. 1992; Rubboli et al. 1994; Court et al. 1999).

Studies of neuromodulatory afferents have shown that the primate thalamus receives modulation from cholinergic, adrenergic, noradrenergic, dopaminergic, serotoninergic, and histaminergic systems (Tables 1, 2, 3, 4, 5). These studies reveal heterogeneous distributions of neuromodulatory axons in the primate thalamus, evidencing non-uniform contributions of each neuromodulatory afferent to information processing across thalamic nuclei. For example, the primate anterior intralaminar nuclei, which are involved in sensory and motor processing, receive dense noradrenergic, serotoninergic, and cholinergic innervation, moderate histaminergic innervation, and sparse adrenergic and dopaminergic innervation; the lateral geniculate nucleus, which is the visual relay nucleus, receives a rather dense cholinergic innervation, moderate histaminergic and serotoninergic innervation, and sparse adrenergic, noradrenergic, and dopaminergic innervation; and the midline nuclei, which are related to limbic and memory circuits, receive dense innervation of all types of neuromodulatory afferents (Lavoie and Parent 1991; Heckers et al. 1992; Rico and Cavada 1998a; Jin et al. 2002; García-Cabezas et al. 2007; Perez-Santos et al. 2021).

**Table 1:** Summary of methods used in studies on the cholinergic innervation of the primate thalamus

Molecule studied	Authors	Genus	Fixation	Postfixation	Technique used	Plane of cutting	Stereotaxic space	Thalamic nomenclature	Parcellation stains	Studied thalamic regions
ChAT	Steriade et al. (1988)	Macaque, Cat	Perfusion (PFA + GTA)	Immersion (PFA)	IMHQ	Coronal	Not specified	Not specified Only major subdivisions	Not specified	Relay and association nuclei
ChAT	Heckers et al. (1992)	Human	Immersion (PFA)	–	IMHQ	Coronal	Not specified	Hirai and Jones (1989)	Nissl, AChE	Whole thalamus
ChAT and vAChT	Rico and Cavada (1998b)	Macaque	Perfusion (PFA)	–	IMHQ	Coronal	YES	Olszewski (1952)	Nissl, AChE, Myelin	Whole thalamus
Nicotinic receptors	Cimino et al. (1992)	Macaque	Unfixed sections	–	Autoradiography	Coronal	Not specified	Olszewski (1952)	Not specified	Whole brain
			Immersion (PFA)	–	In situ hybridization					
Nicotinic receptors	Rubboli et al. (1994)	Human	Unfixed sections	–	Autoradiography	Coronal	Not specified	Not specified Only major subdivisions	Not specified	Whole brain
			Immersion (PFA)	–	In situ hybridization					
Nicotinic receptors	Spurden et al. (1997)	Human	Unfixed sections	–	Autoradiography	Coronal	Not specified	Hirai and Jones (1989)	Not specified	Whole thalamus
Nicotinic receptors	Court et al. (1999)	Human	Unfixed sections	–	Autoradiography	Coronal	Not specified	Hirai and Jones (1989)	AChE, Nissl	Whole thalamus
Nicotinic receptors	Han et al. (2000)	Macaque	Perfusion (PFA)	Immersion (PFA)	In situ hybridization	Coronal	Not specified	Only major subdivisions	Nissl	Whole brain
Nicotinic receptors	Quik et al. (2000)	Saimiri	Frozen-cut sections. Immersion (PFA)	–	In situ hybridization	Coronal	Not specified	Emmers and Akert (1963)	Nissl	Whole brain

*AChE* Acetylcholinesterase, *ChAT* choline acetyltransferase, *GTA* glutaraldehyde, *IMHQ* immunohistochemistry, *PFA* paraformaldehyde, *vAChT* vesicular transporter of acetylcholine

**Table 2:** Summary of methods used in studies on the adrenergic and noradrenergic innervation of the primate thalamus

Molecule studied	Authors	Genus	Fixation	Postfixation	Technique used	Plane of cutting	Stereotaxic space	Thalamic nomenclature	Parcellation stains	Studied thalamic regions
Adrenaline	Mefford et al. (1978)	Human	Unfixed	–	Liquid chromatography	Coronal	Not specified	Only major subdivisions	Fresh brain	Whole brain
PNMT	Rico and Cavada (1998a)	Macaque	Perfusion (PFA)	–	IMHQ	Coronal	YES	Olszewski (1952)	Nissl, AChE, Myelin	Whole thalamus
DßH	Vogt et al. (2008)	Macaque	Perfusion (PFA)	–	IMHQ	Coronal	Not specified	Olszewski (1952)	NeuN, Calbindin, Calretinin	Midline, mediodorsal, and intralaminar thalamic nuclei
NET	Smith et al. (2006)	Macaque	Unfixed	–	Autoradiography	Coronal	Not specified	Only major subdivisions	Not specified	Whole brain
DßH, NET and alpha adrenoceptors	Pérez-Santos et al. (2021)	Macaque	Perfusion (PFA) and unfixed	–	IMHQ and autoradiography	Coronal	YES (DßH and NET data)	Olszewski (1952)	Nissl, AChE, Myelin	Whole thalamus
Beta adrenoceptors	Reznikoff et al. (1986)	Human	Unfixed	–	Autoradiography	Coronal	Not specified	Only major subdivisions	Not specified	Whole brain

*AChE* Acetylcholinesterase, *DßH* dopamine-beta-hydroxylase, *IMHQ* immunohistochemistry, *NET* noradrenaline transporter, *NeuN* neuronal nuclear protein, *PFA* paraformaldehyde, *PNMT* phenylethanolamine N-methyltransferase

**Table 3:** Summary of methods used in studies on the dopaminergic innervation of the primate thalamus

Molecule studied	Authors	Genus	Fixation	Postfixation	Technique used	Plane of cutting	Stereotaxic space	Thalamic nomenclature	Parcellation stains	Studied thalamic regions
DA, TH, and DAT	Sánchez-González et al. (2005)	Human, Macaque	Perfusion (PFA or PFA + GTA)	Immersion (PFA) [human tissue]	IMHQ	Coronal	YES	Olszewski (1952)	Nissl, AChE, Myelin	Whole thalamus
DA and DAT	García-Cabezas et al. (2007)	Human, Macaque	Perfusion (PFA or MBS + GTA)	Immersion (PFA) [human tissue]	IMHQ	Coronal	YES	Olszewski (1952)	Nissl, AChE, Myelin	Whole thalamus
DAT	García-Cabezas et al. (2009)	Macaque, Rat	Perfusion (PFA or PFA + GTA)	Immersion (osmium tetroxide) after IMHQ [only for EM]	IMHQ	Sagittal	YES	Olszewski (1952)	Nissl, AChE, Myelin	Whole thalamus
D2 receptors	Kessler et al. (1993)	Human	Unfixed	–	Autoradiography	Not specified	Not specified	Only major subdivisions	Not specified	Whole brain
D2 and D3 receptors	Gurevich and Joyce (1999)	Human	Immersion (PFA) and unfixed	–	In situ hybridization and autoradiography	Not specified	Not specified	Paxinos (1990)	Nissl, AChE	Whole brain
D2-like receptors	Rieck et al. (2004)	Human	Immersion (only for NPT assessment)	–	Autoradiography and PET	Coronal	Not specified	Mai et al. (1997)	Not specified	Whole brain

*AChE* Acetylcholinesterase, *DA* dopamine, *DAT* dopamine transporter, *EM* electron microscopy, *GTA* glutaraldehyde, *IMHQ* immunohistochemistry, *MBS* sodium metabisulfite, *NPT* neuropathological, *PET* positron emission tomography, *PFA* paraformaldehyde, *TH* tyrosine hydroxylase

**Table 4:** Summary of methods used in studies on the serotoninergic and histaminergic innervation of the primate thalamus

Molecule studied	Authors	Genus	Fixation	Postfixation	Technique used	Plane of cutting	Stereotaxic space	Thalamic nomenclature	Parcellation stains	Studied thalamic regions
Histamine	Lipinski et al. (1973)	Human	Unfixed	–	Enzymatic double-isotope assay	Coronal	Not specified	Only “anterior”, “dorsal” and “pulvinar”	Fresh brain	Whole brain
Histamine	Manning et al. (1996)	Macaque	Perfusion (EDCDI or PFA + EDCDI)	Immersion (EDCDI or PFA + EDCDI)	IMHQ	Not specified	Not specified	Kaas et al. (1978). Only LGN and Pul-LP	Not specified	Visual-related structures
Histamine and H_1_, H_2_, and H_3_ receptors	Jin et al. (2002)	Human	Immersion (EDAC)	Immersion (PFA)	IMHQ, In situ hybridization, and autoradiography	Coronal	Not specified	Hirai and Jones (1989)	Nissl	Whole thalamus
5-HT	Lavoie and Parent (1991)	Saimiri	Perfusion (PFA + GTA)	Immersion (PFA)	IMHQ	Coronal	Not specified	Adaptation of Emmers and Akert (1963) following Olszewski (1952)	Nissl	Whole thalamus
5-HT_1_ receptors	Pazos et al. (1987a)	Human	Unfixed	–	Autoradiography	Coronal	Not specified	Dewulf (1971)	Not specified	Whole brain
5-HT_2_ receptors	Pazos et al. (1987b)	Human	Unfixed	–	Autoradiography	Coronal, horizontal	Not specified	Dewulf (1971)	Not specified	Whole brain
5-HT receptors	Wai et al. (2011)	Human	Immersion (PFA)	–	IMHQ	Coronal	Not specified	Not specified	Nissl, Myelin	Developing thalamus

*5-HT* 5-hydroxytriptamine (serotonin), *EDAC* 1-ethyl-3,3(dimethylaminopropyl)carbodiimide, *EDCDI* l-ethyl-3(3-dimethylaminopropyl)-carbodiimide, *GTA* glutaraldehyde, *IMHQ* immunohistochemistry, *LGN* lateral geniculate nucleus, *LP* Lateral posterior nucleus, *PFA* paraformaldehyde, *Pul* Pulvinar complex

**Table 5:** Summary of methods used in studies on multiple neurotransmitters in the primate thalamus

Molecule studied	Authors	Genus	Fixation	Postfixation	Technique used	Plane of cutting	Stereotaxic space	Thalamic nomenclature	Parcellation stains	Studied thalamic regions
5-HT and DßH	Morrison and Foote (1986)	Macaque, Saimiri	Perfusion (PFA)	Immersion (PFA)	IMHQ	Coronal	Not specified	Emmers and Akert (1963)	Nissl	Visual-related structures
5-HT and DßH	Westlund et al. (1990)	Macaque	Perfusion (PFA)	–	IMHQ	Coronal	Not specified	Only VPL	Not specified	VPL thalamic nucleus
5-HT and DA	Liu and Jones (1991)	Macaque, Felis (cat)	Perfusion (PFA or PFA + GTA)	Immersion (PFA or PFA + GTA)	IMHQ	Coronal	Not specified	Only VPL	Not specified	VPL thalamic nucleus
5-HT, DA, NA	Oke et al. (1997)	Human	Unfixed	–	Liquid chromatography	Coronal	Not specified	Only major subdivisions	Fresh brain	Whole thalamus
ChAT and histamine	Wilson et al. (1999)	Macaque	Perfusion (PFA or PFA + EDCDI) or immersion (EDCDI)	Immersion (PFA or EDCDI) [only some tissue]	IMHQ	Coronal	Not specified	Only lateral geniculate nucleus	Not specified	Lateral geniculate nucleus
DAT, DßH and TH	Melchitzky and Lewis (2001)	Macaque	Perfusion (PFA)	Immersion (PFA)	IMHQ	Coronal, Sagittal	Not specified	Olszewski (1952), Ilinsky and Kultas-Ilinsky (1987)	Nissl	Mediodorsal nucleus
5-HT and orexin	Hsu and Price (2009)	Macaque	Perfusion (PFA)	Immersion (PFA)	IMHQ	Coronal	Not specified	Olszewski (1952)	Nissl, AChE, Myelin	Paraventricular thalamic nucleus
Multiple receptors	Palomero-Gallagher and Zilles (2018)	Human	Unfixed	–	Receptor autoradiography	Coronal	Not specified	Not specified. Only major subdivisions	Nissl-like and myelin	Whole brain

*5-HT* 5-hydroxytriptamine (serotonin), *DA* dopamine, *DAT* dopamine transporter, *DßH* dopamine-beta-hydroxylase, *ChAT* choline acetyltransferase, *EDCDI* l-ethyl-3(3-dimethylaminopropyl)-carbodiimide, *GTA* glutaraldehyde, *IMHQ* Immunohistochemistry, *NA* noradrenaline, *PFA* paraformaldehyde, *TH* tyrosine hydroxylase, *VPL* ventral posterolateral nucleus

Methodological differences among the studies in Tables 1, 2, 3, 4, 5 highlight three main problems in investigating and analyzing the neuromodulatory systems in the primate thalamus. First, the large number of thalamic nuclei that can be identified in the primate thalamus (Jones 2007) makes surveying this region a painstaking process. Second, the use of different nomenclatures for thalamic subdivisions (Olszewski 1952; Hassler 1959; Hirai and Jones 1989; Percheron et al. 1996) adds confusion to the interpretation and discussion of results from different studies [reviewed in detail in García-Cabezas et al. (2023)]. Finally, the use of different experimental approaches, mapping frames, parcellation criteria, and ways to display results across laboratories hampers data extrapolation. For example, apparent differences in the serotoninergic afferents to the human (Oke et al. 1997) and the non-human primate thalamus (Lavoie and Parent 1991) might be due to actual interspecies differences, or to major methodological differences: biochemistry (Oke et al. 1997) *vs.* immunohistochemistry (Lavoie and Parent 1991) or inaccurate nucleus identification due to fresh tissue processing (Oke et al. 1997) *vs.* fine nucleus identification thanks to adjacent Nissl sections (Lavoie and Parent 1991). Therefore, comparison between published results about the thalamus is not always reliable due to terminological and methodological differences between research groups.

Detailed comparisons between the distribution of several modulatory afferents in the thalamus of a given primate species, or between the distribution of a given modulatory afferent in the thalamus of several primate species are needed for deeper understanding of modulatory processing in the thalamus. In this article, we aim to review the different methodological approaches used to map modulatory neurotransmitter systems through the non-human and human primate thalamus. We analyze the pros and cons of each methodological procedure and propose a systematic methodological framework for processing brains of primate species for the study of neuromodulatory afferents to the thalamus, as well as for the analysis of the data obtained. The proposed methodological framework includes recommendations to conduct brain fixation, cutting, sectioning, tissue staining, parcellation of thalamic nuclei, and analysis of markers in a reproducible manner. Our goal is to facilitate comparisons between neuromodulatory afferents to the thalamus across primate species based on the results of different research groups.

## Methodological proposal

### Obtaining brain tissue: human and non-human primates

The anatomical study of *post-mortem* human brains is highly appreciated for providing findings with pathophysiological relevance for clinical neuroscientists. Human brain tissue is relatively easy to obtain thanks to brain banks, but the conditions of its obtention and processing are less controllable and adaptable, compared to brains obtained from laboratory animals (Shepherd et al. 2019). Close collaborations with hospital pathology services and brain donor programs are needed to collect human brains in tightly controlled and specific conditions.

The anatomical study of brains from animal models complements the study of human brains because it allows experimental procedures like tract-tracing of synaptic connections that are not admissible in humans. Non-human primates (for example, *Rhesus* macaques, see Tables 1, 2, 3, 4, 5) are excellent models for anatomical studies in neuroscience because these species' brain structure and connections are close to humans' (Ventura-Antunes et al. 2013; Phillips et al. 2014). In fact, some studies on neuromodulatory afferents to the thalamus have demonstrated striking differences between rodents and primates (García-Cabezas et al. 2009), but rather similar distributions in humans and non-human primates (García-Cabezas et al. 2007).

The European Directive 2010/63/EU (EU 2010) recognizes the need for the use of non-human primates in biomedical research. This directive, however, also states that the use of non-human primates should only be permitted when no alternative methods are available. In this sense, the EU Scientific Committee on Health Environmental and Emerging Risks Final Opinion “on the need for non-human primates in biomedical research, production and testing of products and devices”, updated in 2017, recognized as essential the use of non-human primates in “Learning how complex brains of primates, humans included, are structured and function. Again, NHPs (*non-human primates*) were considered the best model due to their close similarity to humans with regard to brain complexity and function” (SCHEER 2017).

#### Recommendations for selection of primate species

Primate studies increase the clinical applicability of basic research. Studies of non-human primate brains (like macaques) in naive conditions are necessary, because they lay the foundations for later studies of those systems after in vivo experimental manipulations (pharmacological manipulations, animal models of neurological diseases, etc.). Furthermore, these studies also serve as preclinical references for clinical studies. For instance, the discovery of an abundant dopaminergic innervation in the macaque and human thalamus (Sánchez-González et al. 2005; García-Cabezas et al. 2007), one that is expanded compared to rodents (García-Cabezas et al. 2009), pointed out the dopaminergic receptors in the thalamus as potential therapeutic targets in several neuropsychiatric disorders, like depression (Hirvonen et al. 2011). Also, mapping dopaminergic axons in neurotypical macaques (García-Cabezas et al. 2007) was the first step for quantifying dopaminergic axon loss in the thalamus of a progressive model of Parkinson’s disease in a non-human primate (Monje et al. 2020), a study that points to dopaminergic neurodegeneration in the thalamus in Parkinson’s disease, beyond the degeneration of the nigrostriatal system.

The use of human brain tissue is recommended whenever possible because this tissue provides the most valuable results for translational neuroscience. It is advisable to establish collaborations with brain donor programs and human tissue banks to obtain brain tissue in controlled conditions. Controlled processing of brain tissue from the autopsy room to the laboratory bench is necessary for reproducible, reliable, comparable, and significant results.

### Brain fixation

Fixation is a process that allows long term preservation of biological tissues by preventing autolysis and microbial degradation. Fixation is achieved by chemical agents that harden brain tissue and preserve the general three-dimensional structure of cells and extracellular components. The most widely used fixatives in biology and medicine are crosslink fixatives that establish irreversible covalent links between basic amino-acid residues. Aldehydes are typical crosslink fixatives, formaldehyde being the most used for light microscopy, and glutaraldehyde for electron microscopy (Fox et al. 1985; Layton et al. 2019). Formaldehyde provides good overall tissue quality and preserves the antigenicity of proteins, allowing them to react with specific antibodies.

Fixation of brain tissue is necessary for certain neuroanatomical techniques (for example, immunohistochemistry), but it is not recommendable for others (for example, receptor autoradiography) (see Tables 1, 2, 3, 4, 5). Brains are fixed by perfusion or by immersion. Fixation by perfusion yields more uniform fixation than immersion (McFadden et al. 2019). Immersion, although technically simpler, yields non-homogeneous fixation because superficial parts of brains immersed in fixative solutions will fix faster and more strongly than deeper parts, resulting in gradients of fixation that cause gradients in immunogenicity to certain antibodies (McFadden et al. 2019). Perfusion for fixation of non-human primate brains is the rule in most laboratories but it is not commonly used for human brains (see Tables 1, 2, 3, 4, 5). In fact, in most brain banks, human brains are preserved divided into two hemispheres: one hemisphere is fixed by immersion for anatomical studies and the other one is cryopreserved in small, dissected parts for genetic and biochemical studies (Bauer et al. 2016; McFadden et al. 2019). Thus, high-quality human brain tissue fixed by perfusion is not commonly found in brain banks.

*Post-mortem* delay before fixation is another variable that influences tissue quality in human brains, because long *post-mortem* delays before fixation can interfere with immunolabeling (Lavenex et al. 2009; Gonzalez-Riano et al. 2017). Unfortunately, *post-mortem* delays shorter than 2 h for human brain fixation are difficult to achieve due to legal and technical reasons. Aspects that facilitate short *post-mortem* delays are pre-mortem recruitment, coordination with the donor’s family, and access to laboratory facilities within or near the hospital where donors die. Pre-mortem factors such as agonal states (for example, in deaths caused by cancer, cerebrovascular disease, or bronchopneumonia), brain hypoxia, or coma also influence tissue quality (Stan et al. 2006; Monoranu et al. 2009) and therefore should be taken into account for selecting brains in the best possible conditions.

#### Recommendations for brain fixation

Intracardiac perfusion is the gold standard for fixation of non-human primate brains for typical immunohistochemistry processing (for example, to label synthetizing enzymes, or neurotransmitter transporters). The animal is deeply anesthetized with sodium pentobarbital, the heart is reached via surgery, the ascending aorta is cannulated, and the animal is perfused with saline solution (0.9% NaCl) to wash the blood away, followed by a fixative solution. Typical fixative solutions contain 4% paraformaldehyde in 0.1 M, pH 7.3 phosphate buffer (PB) at room temperature, although the composition of the fixative solution will depend on the technique to be used and the molecule to be revealed (see Tables 1, 2, 3, 4, 5). Fixative solutions are perfused using peristaltic pumps or gravity pressure-driven systems. A series of sucrose solutions in PB 0.1 M, pH 7.3 (5-10-20%, 4 °C) can also be perfused after the fixative solution to start cryoprotection.

Intravascular perfusion is also the gold standard for human brain fixation. Brains from human donors are removed in necropsies with the shortest *post-mortem* delay possible and are washed and fixed by intravascular perfusion. In our laboratory, the brain is carefully removed from the skull without damaging the arteries that irrigate brain tissue. Then, the brain is placed with its dorsal surface on the laboratory bench (upside down, best within a mold to preserve brain shape) to expose its ventral surface. Both internal carotid arteries are cannulated. The basilar artery is also cannulated through one of the vertebral arteries, and the other vertebral artery is ligated. The brain is perfused through both internal carotid arteries and the basilar artery with saline solution at room temperature to wash debris and blood, followed by the fixative solution, which is typically 4% paraformaldehyde in 0.1 M, pH 7.3 phosphate buffer (PB) at room temperature. Fixative solutions are perfused using peristaltic pumps or gravity pressure-driven systems. After perfusion, human brains are cut in 1 cm thick slabs using a stereotaxic frame (see below) and the slabs are post-fixed for approximately 24 h. Postfixation is needed in perfused human brains because perfusion-fixation is not quite optimal due to *post-mortem* delays and intravascular blood clots (see Tables 1, 2, 3, 4, 5).

### Brain cutting: stereotaxic spaces and stereotaxic planes

After fixation (and cryoprotection, if that is the case) brains are slabbed in coronal, sagittal, or horizontal planes. Brain slabs are later sliced into thin sections for histological processing. Alternatively, smaller blocks containing the thalamus are separated and sectioned afterwards. Coronal, sagittal, and horizontal planes have been used to delineate thalamic nuclei (Olszewski 1952; Ilinsky and Kultas-Ilinsky 1987; Hirai and Jones 1989) but coronal sections are the most used for the description of neuromodulatory afferents to the thalamus (see Tables 1, 2, 3, 4, 5). Series of sections through the thalamus in the coronal plane provide a general view of all thalamic nuclei, allow for visualization of the medio-lateral dimension (which is particularly interesting in some nuclei like the mediodorsal nucleus, centromedian-parafascicular complex, or ventral posterior nuclei), and ensure visualization of midline nuclei. Parasagittal planes provide better visualization of the antero-posterior dimension of the thalamus, which is of particular interest to identify and delineate ventral motor nuclei. Finally, horizontal planes, which are frequently used in radiology—for example, Li et al. (2018)—, permit visualization of medio-lateral and anteroposterior dimensions.

Independently of the selected plane, brains can be cut in approximate coronal, sagittal, or horizontal planes, or in precise stereotaxic planes (see Tables 1, 2, 3, 4, 5), which are obtained with specific references related to stereotaxic spaces. Stereotaxis was developed in the first half of the twentieth century for precise targeting of brain structures (Rahman et al. 2009) and aims to define spatial coordinates based on anatomical landmarks for reproducible and inter-individual comparable positioning and orientation of brains.

In macaques, the most used stereotaxic space is defined by the orbitomeatal plane. This plane goes through the interaural line (between the tips of the earbars in the stereotaxic apparatus) and the inferior orbital ridges (Olszewski 1952; Szabo and Cowan 1984; Paxinos et al. 2000). Horizontal planes in this stereotaxic space are parallel to the orbitomeatal plane; coronal planes are perpendicular to the horizontal plane and parallel to the interaural line; and sagittal planes are perpendicular to the horizontal and coronal planes.

The most widely used stereotaxic space for human brain surgery and imaging is the space of Talairach and Tournoux (1988), which is defined using the anterior and posterior commissures as anatomical landmarks instead of the orbitomeatal plane. In this space, the line connecting the superior margin of the anterior commissure and the inferior margin of the posterior commissure defines the rostro-caudal axis. Coronal planes are perpendicular to the rostro-caudal axis. Parasagittal planes are perpendicular to coronal planes and parallel to the sagittal plane (through the midplane, in between the hemispheres and through the rostro-caudal axis). Finally, horizontal planes are perpendicular to coronal and sagittal planes.

#### Recommendations for brain cutting

Precise stereotaxic cutting of both macaque and human brains is desirable to obtain thalamic sections in stereotaxic planes, and therefore, to present results in reproducible frameworks, facilitating comparison of data across laboratories. Furthermore, stereotaxic planes facilitate the correlation of *post-mortem* anatomical findings with in vivo imaging studies because brain images are also obtained in predetermined stereotaxic spaces, like the Talairach and Tournoux (1988) space.

Stereotaxic cutting of macaque brains is achieved using stereotaxic frames that are commercially available (Olszewski 1952; Szabo and Cowan 1984). In our laboratory, after perfusion, the head of the non-human primate is separated from the body, the cranial vault is removed with a bone rongeur, and the dura mater is cut to expose the brain. The head is placed in the stereotaxic frame adjusting the ear bars in the external auditory meatuses, and the palate and infraorbital adapters in the right position. The stereotaxic arm, holding a scalpel blade parallel to the coronal plane, is placed in previously measured coordinates of the interaural plane, which is considered the stereotaxic anteroposterior level (AP) 0. The blade is moved down, up, and latero-medio-laterally to cut the brain in the coronal plane. This procedure is repeated in AP = 1, AP = 2, AP = − 1, AP = − 2, etc., to obtain 1 cm slabs—for a more detailed description of comparable procedures performed in other laboratories see Burke et al. (2009). To produce sagittal slabs the same system is followed, with the scalpel in the stereotaxic arm placed in the sagittal plane and moving from rostral to caudal while cutting.

There are no commercially available stereotaxic frames for cutting human brains. In our laboratory, we fabricated a stereotaxic frame for this purpose. The frame consists of a transparent rectangular methacrylate plate with a centered line drawn along the longest axis. A mirror is placed under the methacrylate plate, to visualize the position of the structures facing the plate. The frame holds on its longest sides a series of metal bars perpendicular to the methacrylate plate. These bars leave slots to introduce a blade every centimeter to cut the brain in a plane that is perpendicular to the methacrylate plate, and perpendicular to the line drawn in the methacrylate plate.

To obtain human coronal slabs, the brainstem is separated from the brain and the two hemispheres of the brain are separated by cutting the corpus callosum through the mid-sagittal plane. Then, each hemisphere is placed on the in-house stereotaxic frame with the anterior and posterior commissures aligned with the line drawn in the methacrylate plate following Talairach and Tournoux (1988). Placing the brain like that leaves the coronal plane perpendicular to the line in the methacrylate plate and to the surface of the methacrylate plate (which corresponds to the sagittal plane), while horizonal planes are parallel to the line in the methacrylate and perpendicular to the methacrylate plate itself. Thus, coronal slabs are cut with a blade through the slots in between the metal bars (perpendicular to the line in the methacrylate plane). Coronal plane 0 corresponds to the posterior margin of the anterior commissure.

The above simple methods allow for reproducible cutting within stereotaxic planes of macaque and human brains. Subsequently, block sectioning produces sagittal, coronal, or horizontal sections that are fully comparable with atlases that use the same space. Furthermore, stereotaxic cutting provides precise and accurate anteroposterior, mediolateral, or dorsoventral levels for each processed section, by calculating the separation between the section of interest and the cutting plane (− 1, 0, + 1, + 2… cm), ﻿by counting the number of sections in between and by considering the thickness of each section. This precise anteroposterior, mediolateral, or dorsoventral localization facilitates comparison with output from in vivo imaging techniques, as well as with atlases, or with other studies that also used stereotaxic space.

### Tissue sectioning and preservation

Brain slabs obtained either with or without stereotaxic planes must be sliced into thin sections for histological processing. Slabs of macaque brains are usually sectioned entirely in thin sections that contain whole hemispheres in coronal, sagittal, or horizontal planes. In contrast, when sectioning human brains, prior dissection of blocks containing the region of interest (the thalamus) is usually done, although slabs of whole human brain hemispheres can also be sectioned in one piece.

Before sectioning, brain blocks must be hardened either by freezing or by embedding in wax (paraffin/celloidin). Frozen tissue is sectioned in freezing microtomes or cryostats, and embedded tissue is sectioned in regular rotatory microtomes. Paraffin/celloidin embedding requires tissue dehydration by incubation with increasing concentrations of alcohol and with xylene before wax immersion. Embedding facilitates tissue preservation at room temperature, but freeze sectioning better preserves lipids and protein antigenicity. Also, freeze sectioning allows for easy collection of consecutive serial sections that are preserved in good conditions for years floating in ethylene–glycol antifreeze solution at – 20 ºC.

#### Recommendations for tissue sectioning and preservation

We recommend the use of freeze sectioning for human and non-human brain tissue. Cryoprotection of fixed brain blocks containing the thalamus is advisable before freeze sectioning. Cryoprotection is achieved by immersion in gradually increasing concentrations of sucrose (5, 10, 20, and 30% in 0.1 M, pH 7.3 PB solutions). It is possible to start this process by perfusion of the sucrose solutions as explained for macaque brains in the fixation section.

Several series of brain sections can be collected while sectioning. Each series can be processed to reveal different molecules or stains for thalamic delineation. For example, 10 series of 40 μm thick sections allow for 10 different stains, and each section processed for a particular stain will be 400 μm apart from the next or previous section processed for the same stain. This is key for unbiased quantification as will be explained below. Sections cut in the freezing microtome can be immediately processed or stored at − 20 °C in an ethylene–glycol antifreeze solution in the laboratory tissue bank until further processing.

For techniques that require unfixed tissue (for example, autoradiography), fresh brain bocks are snap frozen without cryoprotection by immersion in isopentane at − 40ºC. Then, blocks are serially sectioned at 20 μm in a large-scale cryostat (at − 20ºC). Sections are mounted on gelatin-coated glass slides and dried under a cold-air stream. Sections attached to glass slides are stored at – 80 ºC until the next processing steps (Zilles et al. 2002; Palomero-Gallagher and Zilles 2018).

### Thalamic nuclear terminology: macaque and human

Over the course of the twentieth century, several thalamic nuclear parcellation schemes were proposed based on microscopic observations (García-Cabezas et al. 2023). Thalamic parcellations in primates are broadly framed in two groups. First, is the Anglo-American School, which is summed up in Walkers’ monography on the primate thalamus (Walker 1938), followed and developed by Olszewski’s atlas of the macaque thalamus (Olszewski 1952), updated for the human thalamus by Hirai and Jones (1989), and revised for ventral motor nuclei by Ilinsky and Kultas-Ilinsky (1987), who adapted this scheme to the human thalamus with stereotaxic coordinates and 3D reconstructions (Ilinsky et al. 2018). The second is the German School, initiated in Cecile and Oskar Vogt´s laboratory (Vogt 1909; Friedemann 1911); expanded and systematized by Hassler, and used in the stereotaxic atlas of the human brain by Schaltenbrand and Bailey (Hassler 1959). The German School thalamic subdivision is more complex than the Anglo-American parcellation, but it was widely used by neurosurgeons due to its inclusion in Schaltenbrand’s human brain stereotaxic atlas (Schaltenbrand and Bailey 1959), whose subsequent edition (Schaltenbrand et al. 1977) is still used nowadays by many neurologists and neurosurgeons, particularly for the subdivisions of the ventral motor nuclei (Hamani et al. 2006; Vassal et al. 2012); [see García-Cabezas et al. (2023) in this issue of Brain Structure and Function for a more detailed analysis of the thalamic nuclear parcellation development].

The Anglo-American School parcellation, simpler than the German School parcellation and directly related to connectivity, has been widely accepted by most basic neuroscientists (examples in Tables 1, 2, 3, 4, 5). In fact, the parcellation of the human thalamus by Hirai and Jones (1989) was adapted to stereotaxic coordinates (Morel et al. 1997; Morel 2007) and has been chosen as the standard nomenclature in the Terminologia Neuroanatomica, which is a consensus terminology published by the International Federation of Associations of Anatomists (FIPAT 2017; Ten Donkelaar et al. 2017).

#### Recommendations for thalamic nuclear terminology

We propose the thalamic nuclear parcellation and terminology of Olszewski (1952) with some modifications (Cavada et al. 1995) for the macaque thalamus, and the parcellation and terminology of Hirai and Jones (1989) for the human thalamus. The choice of these thalamic parcellations from the Anglo-American tradition is grounded on their wide and growing use by primate-focused neuroscientists, on the morphological and functional rationale underlying them, and on their clarity, simplicity, and reproducibility (García-Cabezas et al. 2023).

### Stains for thalamic nuclear parcellation

Delineation of thalamic nuclei requires staining brain tissue to reveal histological characteristics that allow differentiation of thalamic nuclei. Several methods have been used to this end since the late nineteenth century (García-Cabezas et al. 2023), including cresyl-violet staining (Nissl staining), myelin staining, acetylcholinesterase (AChE) histochemistry, and cytochrome oxidase (CO) histochemistry. Each of these stains reveals different morphological characteristics of thalamic nuclei and is of particular interest in delineating specific nuclei.

Nissl staining uses basic dyes that bind to nucleic acids (DNA, RNA) in all brain cells (neurons, astrocytes, microglia, oligodendrocytes, endothelial cells…). As a result, Nissl staining labels the cell nuclei and cell bodies of neurons, as well as the nuclei of glial cells, endothelium and ependyma. Therefore, Nissl staining reveals cell density, neuron cell body size and shape, cell nucleus size, nucleolus, euchromatin and heterochromatin content in the cell nucleus…; in sum, the cytoarchitecture of the stained brain region. Nissl staining is particularly useful in identifying thalamic nuclei or subnuclei with characteristic neuron body sizes (for example, the paracentral nucleus, the magnocellular divisions of the mediodorsal nucleus and the ventral anterior nucleus, the parvocellular layers of the lateral geniculate nucleus, or the densocellular part of the mediodorsal nucleus). Cytoarchitecture has been widely used to delineate thalamic nuclei in primates (Olszewski 1952; Hassler 1977; Ilinsky and Kultas-Ilinsky 1987; Stepniewska et al. 1994) and therefore is highly useful for identification of thalamic nuclei (Tables 1, 2, 3, 4, 5).

Myelin staining allows for visualization of myelinated axons, and therefore, reveals thalamic myeloarchitecture. The most common myelin stain uses Gallyas technique (Gallyas 1979). Myelin staining has been used as a reference in some atlases (Olszewski 1952; Hirai and Jones 1989) and facilitates the identification of internal and external medullary laminae, the reticular nucleus, some poorly myelinated nuclei -for example, midline nuclei, the parvocellular subdivision of ventral posteromedial nucleus-, and some nuclei encapsulated in a thin layer of myelin—for example, centromedian.

AChE histochemistry reveals the activity of the so-called enzyme, providing information on thalamic chemoarchitecture. AChE has been used in some atlases for delineating thalamic nuclei (Hirai and Jones 1989), and it has been proven to distinguish some differentially connected thalamic sectors more reliably than Nissl staining or CO preparations (Stepniewska et al. 1994; Cavada et al. 1995), including subdivisions within the ventral motor thalamus and within the mediodorsal nucleus.

CO histochemistry (Wong-Riley 1979) allows visualization of the activity of that mitochondrial enzyme. CO histochemistry has long proven to be useful to differentiate segregated pathways in the visual cortex (Shipp and Zeki 1985) and has been used to distinguish thalamic territories in the primate thalamus (Jones et al. 1986; Hirai and Jones 1989; Stepniewska et al. 1994). This staining can distinguish the “rod domain” and the “matrix domain” in ventral posterior nuclei (Jones et al. 1986; Rausell and Jones 1991).

**Fig. 1:**
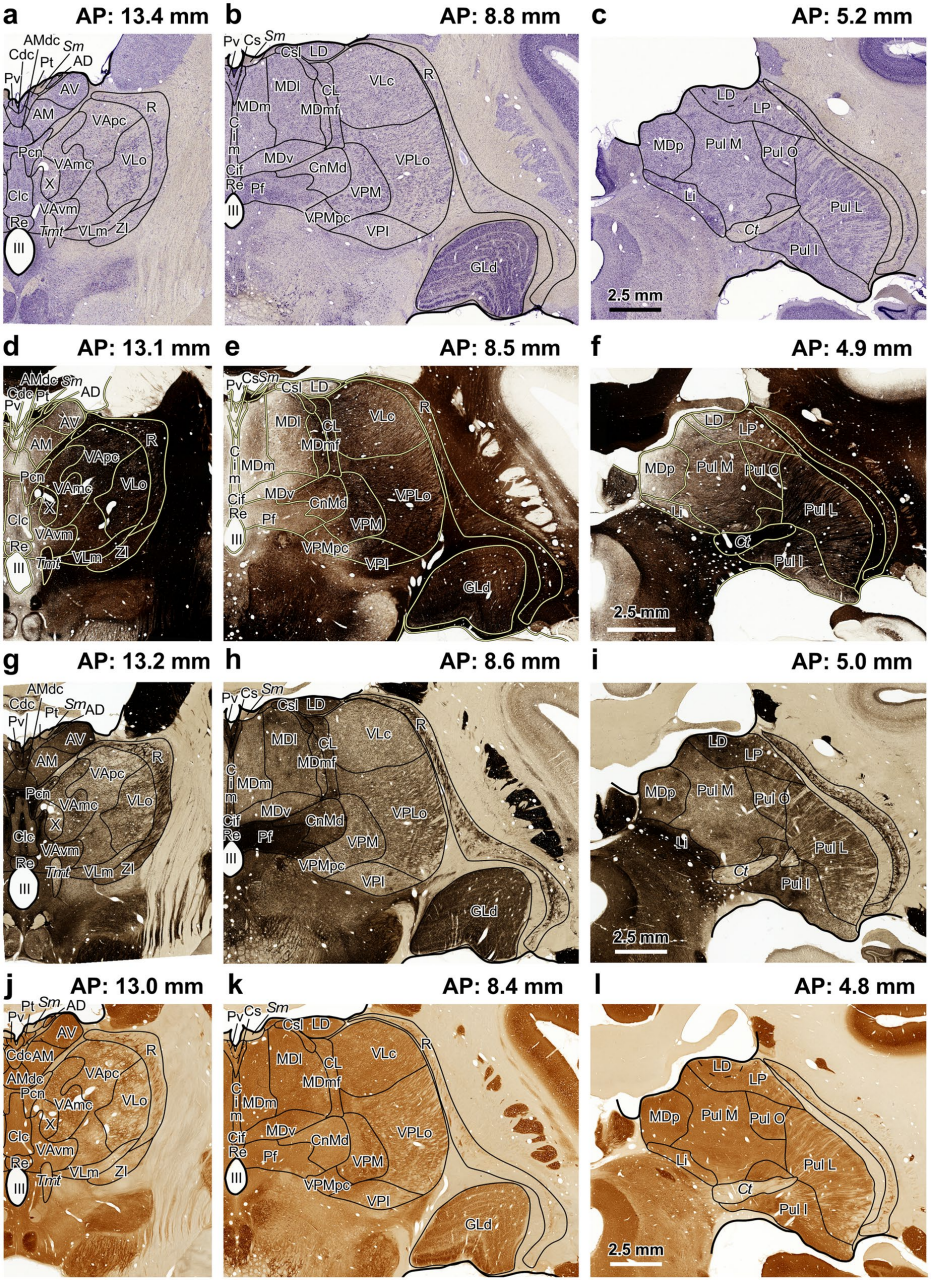
Coronal sections through the macaque thalamus processed with the recommended parcellation stains. **a**–**c**, sections processed for Nissl staining; **d**–**f**, sections processed for myelin staining; **g**–**i**, sections processed for acetylcholinesterase histochemistry; **j**–**l**, sections processed for cytochrome oxidase histochemistry. Each calibration bar applies to the three pictures in the row. Abbreviations: see Table 6

**Fig. 2:**
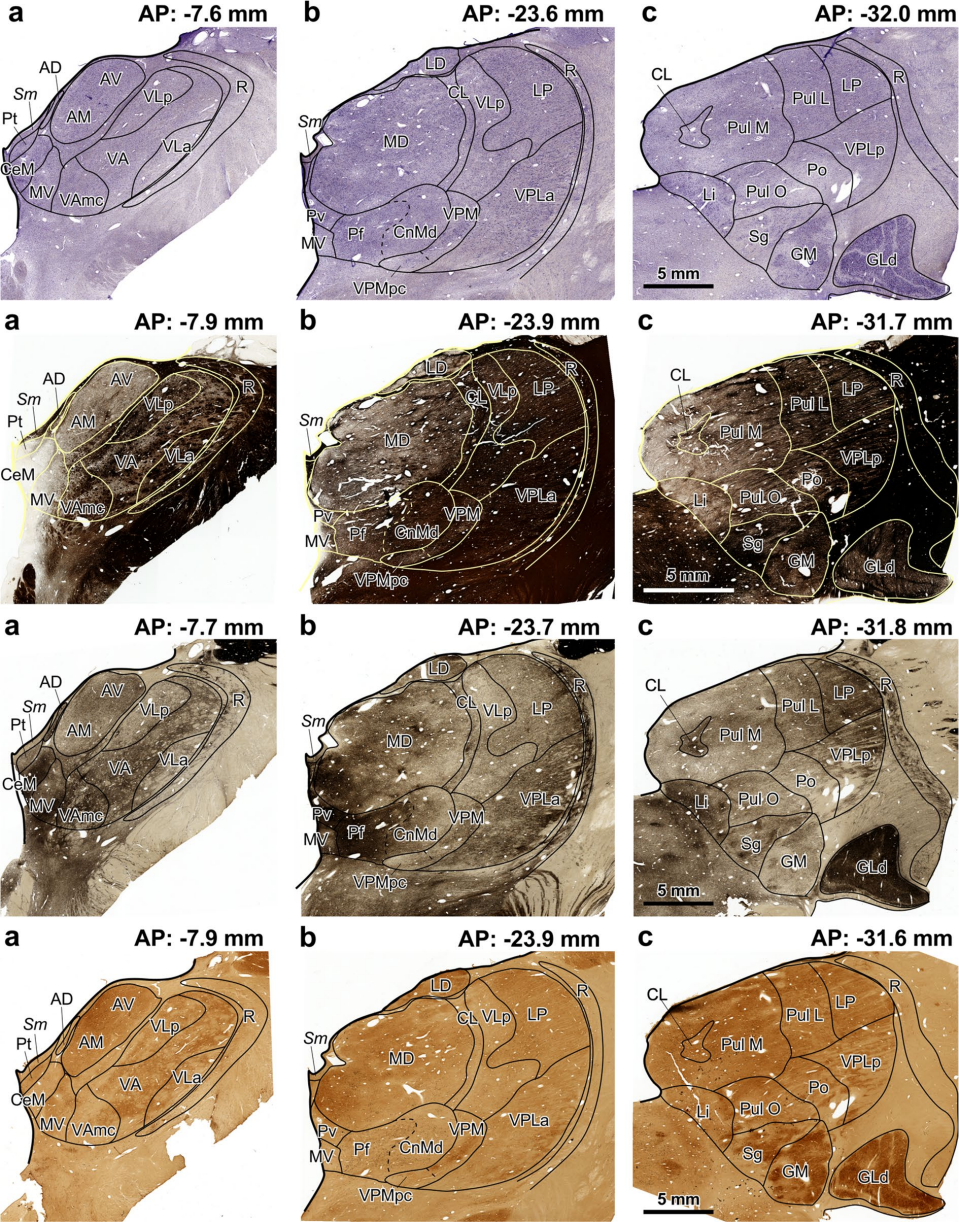
Coronal sections through the human thalamus processed with the recommended parcellation stains. **a**–**c**, sections processed for Nissl staining; **d**–**f**, sections processed for myelin staining; **g**–**i**, sections processed for acetylcholinesterase histochemistry; **j**–**l**, sections processed for cytochrome oxidase histochemistry. Each calibration bar applies to the tree pictures in the row. Abbreviations: see Table 6

As illustrated in Figs. 1 and 2 (see Table 6 for abbreviations in these Figures), each of the above stains or a combination of two or three of them are suitable for identifying specific thalamic nuclei. For instance, Nissl and AChE stains are particularly valuable for studying the mediodorsal nucleus, while CO staining is especially useful in studying the ventral posterior nuclei (Figs. 1, 2, Table 6).

**Table 6:** Abbreviations used in Figs. 1 and 2

AD	Anterodorsal nucleus	Po	Posterior nucleus^b^
AM	Anteromedial nucleus	Pt	Paratenial nucleus
AMdc	Anteromedial nucleus densocellular part^a^	Pul I	Inferior pulvinar nucleus^a^
AP	Stereotaxic anteroposterior level	Pul L	Lateral pulvinar nucleus
AV	Anteroventral nucleus	Pul M	Medial pulvinar nucleus
Cdc	Central nucleus–densocellular part^a^	Pul O	Oral pulvinar nucleus
CeM	Central medial nucleus^b^	Pv	Paraventricular nucleus
Cif	Central nucleus–inferior part^a^	R	Reticular nucleus
Cim	Central nucleus–intermediate part^a^	Re	Nucleus reuniens^a^
CL	Central lateral nucleus	Sg	Suprageniculate nucleus^b^
Clc	Central nucleus–latocellular part^a^	*Sm*	Stria medullaris
CnMd	Centromedian nucleus	*Tmt*	Mamillothalamic tract^a^
Cs	Central superior nucleus^a^	VA	Ventral anterior nucleus^b^
Csl	Central nucleus–superior lateral part^a^	VAmc	Ventral anterior nucleus–magnocellular part
*Ct*	Corticotectal tract^a^	VApc	Ventral anterior nucleus–parvocellular part^a^
GLd	Dorsal lateral geniculate nucleus	VAvm	Ventral anterior nucleus–ventromedial part^a^
GM	Medial geniculate nucleus^b^	VLa	Ventral lateral nucleus–anterior part^b^
LD	Lateral dorsal nucleus	VLc	Ventral lateral nucleus–caudal part^a^
Li	Limitans nucleus	VLm	Ventral lateral nucleus–medial part^a^
LP	Lateral posterior nucleus	VLo	Ventral lateral nucleus–oral part^a^
MD	Mediodorsal nucleus^b^	VLp	Ventral lateral nucleus–posterior part^b^
MDl	Mediodorsal nucleus–lateral sector^a^	VPI	Ventral posterior inferior nucleus^a^
MDm	Mediodorsal nucleus–medial sector^a^	VPLa	Ventral posterior lateral nucleus–anterior part^b^
MDmf	Mediodorsal nucleus–multiformis sector^a^	VPLo	Ventral posterior lateral nucleus–oral part^a^
MDv	Mediodorsal nucleus–ventral sector^a^	VPLp	Ventral posterior lateral nucleus–posterior part^b^
MDp	Mediodorsal nucleus–posterior sector^a^	VPM	Ventral posterior medial nucleus
MV	Medioventral nucleus^b^	VPMpc	Ventral posterior medial nucleus–parvocellular part
Pcn	Paracentral nucleus^a^	X	Area X^a^
Pf	Parafascicular nucleus	ZI	Zona incerta^a^

^a^Abbreviations used only for the macaque thalamus

^b^Abbreviations used only for the human thalamus

#### Recommendations for thalamic nuclear parcellation

We recommend performing parcellation stains on sections adjacent to those processed to reveal the molecule of interest (neurotransmitters, enzymes, transporters, receptors…) when studying neuromodulatory afferents to the thalamus. The use of Nissl staining and AChE, at least, is advisable to reliably identify and delineate human and macaque thalamic nuclei. Other stains, like Myelin or CO, facilitate the identification of specific thalamic structures and can complement Nissl and AChE stains (Figs. 1, 2, Table 6). Thalamic nuclei are identified and delineated in Nissl-stained sections, double-checking with sections processed for other stains (for example, AChE and Myelin).

### Histological processing of neuromodulatory systems

Neuromodulatory afferents to the thalamus are studied by detection of neurotransmitters, synthetizing and degradation enzymes, transporters, and/or receptors. Several techniques have been used to reveal these molecules, including biochemical detection of neurotransmitters (Oke et al. 1997; Pifl et al. 2012, 2013), autoradiography with tritiated ligands that bind neurotransmitter transporters (Smith et al. 2006) or neurotransmitter receptors (Jin et al. 2002; Pérez-Santos et al. 2021), in situ hybridization to reveal the distribution of mRNAs coding for neurotransmitter receptors or related enzymes (Jin et al. 2002), or immunohistochemistry to reveal neurotransmitters (Jin et al. 2002; García-Cabezas et al. 2007), their synthetizing and degradation enzymes (Rico and Cavada 1998a; García-Cabezas et al. 2007; Pérez-Santos et al. 2021), or neurotransmitter transporters (García-Cabezas et al. 2007; Pérez-Santos et al. 2021) (see also Tables 1, 2, 3, 4, 5). Spatial transcriptomics is a modern approach to analyze the expression of thousands of genes on brain tissue; it is particularly interesting compared to non-spatial transcriptomics because it includes topographical information, which is critical in studies of the primate thalamus as this is composed of tens of nuclei of different sizes and shapes. Nevertheless, to date, few spatial transcriptomic studies are available on the primate thalamus (Chen et al. 2022).

#### Recommendations to label axons

The staining method selected to reveal axons of neuromodulatory systems depends on several factors, including the neurotransmitter to be studied. Nevertheless, if choice is possible, we recommend using immunohistochemistry to reveal modulatory axons because it can provide fine morphological descriptions of the axons and their terminals, as well as observation of specific distribution patterns within small thalamic regions (Lavoie and Parent 1991; García-Cabezas et al. 2007; Pérez-Santos et al. 2021). It also allows for complementary electron microscopy studies to demonstrate the type of synapse formed by the labeled axons and their postsynaptic profiles (Parent and Descarries 2008; García-Cabezas et al. 2009) (See Tables 1, 2, 3, 4, 5).

Immunohistochemical processing to reveal neurotransmitters, specific transporters, or synthetizing enzymes should consider several factors. First, the specificity of the primary antibody. Second, the conditions specified by the antibody manufacturer (for example, the use of a specific fixative, paraffin embedding or freeze sectioning without embedding, etc.). Third, the need for endogenous peroxidase inactivation, if the development method is based on peroxidase reaction. Fourth, the need of antigen retrieval, if the epitope detected by the antibody is masked by the aldehyde fixation. In our experience antigen retrieval is the most important step to reveal molecules in axons reliably, in particular in human brain tissue. Omitting this step may result in an absence or virtual absence of immunostained axons. Fifth, the concentration of detergent (for example, Triton X-100) in the incubation solutions to improve penetration of the antibody in the thickness of the tissue, as well as to permeate cell membranes. Sixth, the methods to visualize labeled molecules (fluorescence is optimal for double or triple labeling, while methods based on DAB-peroxidase reaction guarantee the long-time durability of the staining). Finally, positive and negative controls should be added. Positive controls usually consist of processing sections containing brain regions in which the distribution of the molecule of interest has been previously studied (for example, striatum for dopamine transporter). Negative controls consist in substituting primary antibodies with sera from the species in which they had been raised. Also, if the molecule of interest is only expressed by small groups of cells (for example, noradrenergic or serotoninergic brainstem cell groups), a series covering the whole brain and brainstem can be processed to prove that cell bodies are only stained in regions where neurons of the neuromodulatory system have been previously described.

#### Recommendations for detecting receptors

Neurotransmitter receptors can be studied by receptor binding, in situ hybridization, or immunohistochemistry.

Receptor binding involves using labeled ligands that specifically bind to the receptors of interest. Usually, ligands are labeled with radioactive isotopes, like tritium, and are revealed by means of autoradiography. Specific methodology for receptor binding protocols and autoradiography can be found in the articles by Zilles et al. (2002) or by Palomero-Gallagher and Zilles (2018). Quantitative autoradiography has two main advantages: it allows quantification of receptor concentrations and provides information about active receptors (excluding the internalized or inactive receptors).

To perform quantitative autoradiography, the brain sections incubated with radioactive ligands are exposed to radiation-sensitive films for several weeks. Then, the films are developed to obtain autoradiograms. For quantitative autoradiography, exposure of brain tissue to radiation-sensitive films should also include scales of known radioactivity concentrations to obtain a calibration curve (Zilles et al. 2002; Palomero-Gallagher and Zilles 2018).

In situ hybridization is an alternative to quantitative autoradiography for detecting the expression of receptors (see Tables 1, 2, 3, 4, 5). Contrary to receptor binding, in situ hybridization does not provide functional information because it reveals mRNA transcripts but not the actual functional receptors present in cell membranes. Also, in situ hybridization does not usually provide quantitative results. However, this technique can be combined with immunohistochemistry to reveal the identity of receptor-expressing cells (Gurevich and Joyce 1999).

In situ hybridization can be performed in fixed or unfixed tissue and involves designing specific probes for the transcripts of interest. Probes can be cDNA, cRNA, and synthetic oligonucleotide probes. There are several labeling methods to detect probes after incubation, including radioactive isotopes, fluorescent dyes, or conjugation of the probe with biotin for development with avidin–biotin complex conjugated with peroxidase or any other enzyme. In situ hybridization protocols must take into account factors that influence effective hybridization during the incubation step, such as the monovalent cation concentration, melting temperature (which will depend on the guanine-cytosine content of the probe), or additional reagents that increase the access of the probe to the target mRNA, such as detergents. Positive controls may include probes against molecules expressed in most mammal cells (for example, actin) while negative controls include incubation with the opposite sense riboprobe, or with a scramble probe (Jensen 2014; Chu et al. 2019).

Receptors in brain tissue can also be detected by immunohistochemistry [for example, Wai et al. (2011)]. However, neurotransmitter receptor immunohistochemistry requires extreme care with regard to antibody specificity, because on-tissue specificity checking is less straightforward than that of other markers for neuromodulatory neurotransmitter axons. In fact, some commercial antibodies for neurotransmitter receptors have proven to be unspecific (Jensen et al. 2009).

### Analysis of neuromodulatory systems across thalamic nuclei

The molecule of interest (neurotransmitter, enzyme, transporter, receptor…) revealed on brain tissue is analyzed qualitatively and/or quantitatively depending on the technique used and the goal of the investigator.

Qualitative analyses comprise the study of the cellular and intracellular location of the molecule of interest (neuron somata, axons, dendritic spines, neuron nuclei, neuron cytoplasm, neuron membrane, astrocytes…), including its distribution across thalamic nuclei: In which nuclei of the thalamus is the molecule of interest found? Is it expressed with comparable density in all thalamic nuclei? Is it homogeneously distributed within a given nucleus? etc. are only a few of the questions that can be asked.

Quantification of molecules of interest across thalamic nuclei can be tackled in several ways, ranging from quantification of the neurotransmitter itself by means of biochemical methods (Mefford et al. 1978; Oke et al. 1997; Pifl et al. 2012, 2013) to quantification of the length of specific immunoreactive axons or axon densities using biased or unbiased methodologies. Oversimplifying, unbiased quantification methods are those that quantify the molecule or the cellular structure of interest in sampling sites selected randomly, uniformly, and systematically (Howard and Reed 2018). In neuroanatomy, unbiased quantification usually refers to stereological estimations.

Studying the thalamus qualitatively and quantitatively poses different challenges to those of studying other brain structures. For example, the cellular distribution in the striatum (caudate nucleus and putamen) is quite homogeneous (Del Rey and García-Cabezas 2022), while neurons and glial cells in the cerebral cortex are organized in layers, which demand clear identification (García-Cabezas et al. 2020). In the thalamus, neurons are distributed across nuclei, and this makes the identification of nuclear boundaries the critical step for qualitative and quantitative studies. Also, some nuclei are small and others are heterogeneous, composed of sectors with different cellular distributions. Thus, sampling for studying thalamic nuclei must be carefully planned in advance. The following recommendations rely mostly on the authors’ experience on studying the non-human and human primate thalamus.

#### Recommendations for qualitative analysis

Density tables—for example, Jin et al. (2002)—or axon distribution maps—for example, García-Cabezas et al. (2007)—are usually provided to convey the results of qualitative analyses of the molecule of interest. In particular, distribution maps are demonstrative and transmit information in simple, visual, and intuitive ways. In the thalamus, the maps should include serial sections because of the irregular shapes of each thalamic nucleus in 3D.

Maps of labeled axons are drawn using camera lucida (Lavoie and Parent 1991; Rico and Cavada 1998a), or a microscope attached to a computer (García-Cabezas et al. 2007; Hsu and Price 2009). Alternatively, a series of filters applied to microphotographs transforms each pixel into black (signal) or white (background) based on pixel luminance and yields monochromatic images (Pérez-Santos et al. 2021). The transformation of the original images to black and white images providing biological information is called binarization or segmentation. The use of interactive machine learning software for automatic segmentation of biological images—for example, Ilastik (Berg et al. 2019)—may help create axon distribution maps and reduce the time employed to draw them.

Receptor maps are obtained from radiation-sensitive films that have been exposed to brain sections incubated with specific tritiated ligands (see above). These films are developed to obtain autoradiograms. Receptor maps are obtained from autoradiograms in three steps. First, digitization of the autoradiogram film in tightly controlled conditions of light and exposure. Second, transformation of the digitized autoradiograms to images in which gray values are linearly related to receptor concentration (“linearized images”). This transformation is possible thanks to calibration curves obtained from the gray values of the known radioactivity scale in the autoradiograms. Finally, linearized images are pseudocolored by assigning a spectrum of colors to equally spaced gray value ranges. Pseudocolored images will constitute the final receptor map. The color bar that associates each color with a range of receptor concentrations can be displayed next to the maps. For a thorough description of the whole process see Zilles et al. (2002) or Palomero-Gallagher and Zilles (2018).

The borders of thalamic nuclei identified in adjacent or nearby sections processed for parcellation stains are transferred to the maps. References such as the brain ventricles and blood vessels are used as fiduciary marks to transfer nuclear boundaries between sections.

#### Recommendations for quantitative analysis.

Neuromodulatory axons innervating the thalamus are quantified by stereological techniques or optical density measurements.

Stereological estimation of axon length density using isotropic surface probes (Mouton et al. 2002) allows unbiased estimations of axon length or axon density in the thalamic nuclei. This technique has been previously employed to assess axon length densities of subcortical neuromodulatory afferents to the thalamus in non-human primates (Monje et al. 2020). Stereology requires a minimum of 5 to 10 sections for each region of interest (each thalamic nuclei or nuclear group). The greatest strength of stereology is providing results that are reproducible in any laboratory, with an error that can be calculated. This error strongly depends on the number of observations in the random sampling sites (Larsen et al. 1998; Evans et al. 2004). Thus, we recommend the use of stereology whenever it is possible (i.e., if an adequate number of sections is available). The size and shape of thalamic nuclei varies from large and rounded nuclei, like the mediodorsal, to the small and elongated paracentral. Also, their shape in brain sections depends on the plane of cutting. Thus, in stereological studies, the most appropriate distance between sections and framing counts in each section for obtaining the adequate sampling fraction will vary depending on the plane of cutting, as well as on the size and morphology of the studied nucleus: for example, sampling sites in large ovoid nuclei like the mediodorsal nucleus can be separated by a larger distance than sampling sites in thin, small, curved nuclei, like the paracentral nucleus. If stereology is not possible, other methods exist, but they do not guarantee unbiased quantification.

The density of specific neuromodulatory innervation in brain regions other than the thalamus, like the striatum, is often measured by optical density in immunostained tissue (Blesa et al. 2012). However, background staining of primate thalamic nuclei is heterogenous, and could have a major influence on the measured optical density. For this reason, direct measurements of optical density in the immunostained tissue are not advisable in the primate thalamus. The optical density of segmented maps (monochromatic images that display axons in black and the background in white) is an alternative to be considered (Pérez-Santos et al. 2021). Another approach would be to measure the percentage of the area occupied by the axons in microphotographs of the processed tissue, taken with an optic microscope in uniform random sampling sites—for example, outside the thalamus in Zikopoulos et al. (2018).

Quantification of receptor concentrations can be estimated from densitometry on the linearized images (see above), if specifications of the ligand (specific activity of the ligand, dissociation constant of the ligand), ligand concentration during the incubation protocol, and characteristics of the autoradiogram and of the linearization process are known (Zilles et al. 2002; Palomero-Gallagher and Zilles 2018).

Finally, autoradiographic quantification of molecules in the thalamus should also take quenching into account. Quenching is a physical process that consists in blocking the beta emission of tritiated ligands by brain tissue components, particularly myelin. Thus, quenching can strongly affect the apparent distribution and quantification of radioactivity concentrations measured in myelin-rich brain regions (Zilles et al. 1990), like some thalamic nuclei. Quantitative data affected by quenching can be corrected by means of autoradiographic efficiency coefficients (Zilles et al. 1990; Pérez-Santos et al. 2021).

### Public repository of data on neuromodulatory afferents to the primate thalamus

Open access data are gaining value and importance in the scientific community because it makes knowledge accessible to any researcher in any laboratory of the world. In this sense, a public repository to make the data on subcortical neuromodulatory afferents to the thalamus available would contribute to disseminating this information to neuroscientists that are not experts on the thalamus (for example, as a reference in neuroimaging studies). The use of comparable and reproducible methodologies to produce the data shared in the repository would be desirable and help the interpretation of the data provided.

Two examples of public repositories containing human and non-human primate brain maps are the Allen brain map webpage [https://portal.brain-map.org Hawrylycz et al. (2012); Miller et al. (2014)] and the “brainmaps.org” webpage [http://brainmaps.org/index.php Mikula et al. (2007); Jones et al. (2011)]. Although “brainmaps.org” is not a thalamus-exclusive repository, it is a fantastic guide for a thalamus-focused repository. On “brainmaps.org” many human and non-human brain slices, processed for different stains in stereotaxic planes, are available. The thalamus expert Edward Jones (Jones 2007) participated in the creation and promotion of this platform; thus, identification of the various thalamic nuclei in the slices available in this repository is well-founded and reliable.

The importance of public data and tissue image availability does not only apply to current studies; on the contrary, old material from animal brain experiments that nowadays are unaffordable must be kept and preserved, since modern approaches to old material can provide modern valuable data—see for instance Spadory et al. (2022). Preserving old material from expensive and difficult-to-reproduce experiments is part of the responsible use of animals in research and knowledge preservation. This is particularly important regarding neuroscience work with non-human primates, which is becoming increasingly rare, making earlier material still more valuable. Thus, institutions must commit to keeping and preserving research materials, as well as to making them available to scientists all over the world.

### The indispensable role of microscopic neuroanatomy in contemporary studies of the primate thalamus

Microscopic neuroanatomy is one of the most powerful tools for studying and understanding the nervous system. Through the integration of cytoarchitecture, myeloarchitecture, chemoarchitecture, synaptic connections, and gene expression in brains of different species (comparative neuroanatomy), across several stages of life cycles (comparative neurodevelopment and aging), and in neurotypical and pathological brains, neuroanatomy provides a view of brain circuits (paths followed by axons, targets of pathways, postsynaptic elements, receptors in postsynaptic cells, etc.) and brain architecture (neuron density, dendrite length and branching, neuron biochemical phenotype) that transcends structure to gain insights into function (Barbas 2004). Thus, neuroanatomy, by unveiling brain architecture at the molecular, cellular, and synaptic levels, directs the design of electrophysiological experiments, provides data to build neural network models for in silico experiments, establishes criteria for homology hypotheses on brain regions across species, and fills the gap between individual genotypes and behavioral phenotypes (Bohland et al. 2009).

The power of neuroanatomy relies on the high level of resolution provided by neuroanatomical techniques that encompass from subcellular to mesoscale levels. Importantly, neuroanatomical techniques are the only methods that allow for visualization and quantification of synapses by means of electron microscopy (Peters et al. 1976). Also, the study of intact brain tissue allows for integrating scales of resolution like cellular and mesoscale levels. For instance, the scarcity of axons labeled for the dopamine transporter in relay nuclei of the primate thalamus suggests that dopaminergic modulation is not relevant for early sensory processing (García-Cabezas et al. 2007). Other techniques broadly used in contemporary neuroscience, like neuroimaging, do not have enough resolution to be a substitute for neuroanatomical techniques (Paus 2018) or, like genome wide analysis techniques (Nowakowski et al. 2017), must dissociate brain tissue thereby losing the ability to integrate different scales.

Another contribution of neuroanatomy is the clarification and foundation of the concept of homology, a key conceptual tool for data extrapolation across species. According to the classical evolutionary concept of homology, two brain structures are homologues in two different species when both structures have evolved from the same structure in a common ancestor (Wagner 1989). More recently, genoarchitecture through in situ hybridization on whole embryos has allowed identification of progenitor domains that constitute the common *bauplan* of the neural tube for all vertebrate species. Accordingly, two brain structures in two vertebrate species are homologues if they originate in progenitor domains that occupy the same position in the vertebrate *bauplan* (building plan)*.* This is the neuroanatomical basis of field homology (Puelles and Medina 2002), that is used for extrapolating data from rodents to primates (García-Cabezas et al. 2022), as well as from non-human primates to humans.

Despite the power of neuroanatomy, the great intricacy of the nervous system still poses challenges that cannot be fully addressed by the sole use of neuroanatomical techniques. For instance, brain circuits are composed of countless synapses that cannot be labeled, scoped, identified, and quantified in an entire thalamic nucleus, not to mention in entire brains. Also, brain structure and connections vary across species, strains, individuals, and over the life cycle, adding levels of complication that must be taken into account in experimental designs. Despite these challenges, the brain can be tackled by means of well-designed neuroanatomical experiments that run systematic and exhaustive analyses of small representative regions in structures of interest (like thalamic nuclei) at different levels of scale. Specially designed neuroanatomical studies lead to the identification of patterns and regularities through efficient estimations of quantitative neuroanatomical features rather than by trying to fully reconstruct the structure of interest (DeFelipe 2015). In summary, the explanatory power of microscopic neuroanatomy, associated to clinical, electrophysiological, and behavioral data are paramount when exploring the nervous system, because it can integrate several scales and levels of organization across different species, different states of health and disease, and different stages of the life cycle (DeFelipe 2022).

### Challenges in the neuroanatomical study of the thalamus

The thalamus, composed of a variety of nuclei with great connectional diversity, constitutes one of the most heterogeneous brain regions. Thalamic neurons send manifold projections to the cerebral cortex and other structures, like the striatum and the amygdala. Also, thalamic neurons receive inputs from subcortical sources (sensory systems, hypothalamus, amygdala…), from the cerebral cortex, and from subcortical neuromodulatory afferents (Jones 2007). Neuroanatomy is indispensable for studying neuron assemblage into the diverse thalamic nuclei that, located close to each other, form a highly heterogeneous construction.

Regarding neuromodulatory afferents to the primate thalamus, neuroanatomy shows the distribution of neurotransmitter molecules, neurotransmitter transporters etc. across thalamic cells and nuclei. Preserving the structure of brain tissue is key to obtaining contextual information about the thalamic circuits influenced by those afferents. A good example of integration of cytoarchitecture, myeloarchitecture, chemoarchitecture, and connections in intact tissue provided by neuroanatomy pertains to the midline nuclei. In primates, midline nuclei present low myelin concentrations [Figs. 1 and 2; Olszewski (1952)], high AChE enzymatic activity [Figs. 1 and 2; Olivier et al. (1970)], high densities of serotonin axons (Lavoie and Parent 1991), high density of dopamine axons and low density of dopamine transporter immunoreactive axons (García-Cabezas et al. 2007), high density of noradrenaline axons (Pérez-Santos et al. 2021), and high density of histamine axons (Jin et al. 2002). Thus, midline nuclei are strongly modulated by subcortical neuromodulatory systems; they are also poor in myelin, which is a potent inhibitor of synaptic plasticity (Akbik et al. 2013). Therefore, thalamocortical neurons in midline nuclei may respond differently to the same stimulus depending on the activity of all those neuromodulatory afferents. Tract tracing studies in macaques show that midline nuclei are strongly connected with limbic and association cortices (Vogt et al. 1987; Barbas et al. 1991; Webster et al. 1993; Cavada et al. 1995), as well as with the limbic striatum (Giménez-Amaya et al. 1995) and the amygdala (Timbie et al. 2020). Thus, the varied biochemical modulation of thalamic midline nuclei and their limbic circuits may confer more flexibility in the responses of midline neurons compared to neurons in other thalamic nuclei.

Finally, the species of choice is relevant for neuroanatomical studies in the thalamus because the layout of thalamic circuits differs between primates and rodents. For studies on development (Botella-López et al. 2019) or on specific mechanisms [for example, the effects of protein disfunction (Tottene et al. 2019), or the electrophysiology of thalamic neurons (Castro-Alamancos and Calcagnotto 2001)] rodents may be the species of choice, since small animals like mice are easier to manipulate. However, to investigate human brain function and in particular the thalamus, primates are the most suitable model for several reasons: some thalamic nuclei are expanded in primates [for example, the multiformis sector of mediodorsal nucleus, the pulvinar nucleus, and several ventral nuclei; Jones (2007)] and do not have clear homologues in rodents. Also, the population of GABAergic interneurons is significantly expanded in the thalamic nuclei of primates compared to rodents (Jones 2007), adding another variable to primate thalamic circuits because they participate in elaborate synaptic arrangements (Kultas-Ilinsky and Ilinsky 1991; Ilinsky et al. 1999; Timbie et al. 2020). Thus, neuroanatomical studies of the thalamus performed in human and non-human primates will provide significant data to help understand human brain function and behavior with clinical insight.

## Conclusion

In this article, we propose reproducible methodological approaches for designing and developing neuroanatomical studies on the neuromodulatory afferents to the primate thalamus. These methodological approaches would allow for comparison of data obtained from different laboratories. The proposal in this paper includes recommendations for standardizing the quality of brain tissue, the use of standard stereotaxic planes, alternatives for tissue sectioning and preservation, standardized thalamic nuclear parcellation and terminology based on the Anglo-American School, the most useful stains to delineate the thalamic nuclei, the many possibilities to process brain tissue to reveal neurotransmitter-related molecules, potential analyses to be done, and, finally, the need for a universal public repository that would make the data available to specialized and non-specialized researchers.

Neuroanatomical work, in the thalamus and elsewhere, when performed with rigorous standards as proposed here, has the strength to yield accurate, contextual, and multiscale information on the brains of different species. Reliable neuroanatomical data are unambiguous, precise, and overarching, permitting solid conceptual development and breakthroughs in brain understanding.

## Data Availability

All materials used in the manuscript can be requested to the authors.
